# Cross-cultural adaptation, validity, and reliability of the Wexner questionnaire in patients with functional constipation in an Iranian population 

**Published:** 2021

**Authors:** Fatemeh Majidirad, Mohammad-Reza Hadian, Hossein Asl Soleimani, Shohreh Jalaie, Tannaz Ahadi, Roxana Bazaz Bebahani, Hossein Bagheri

**Affiliations:** 1 *Physical Therapy Department, Faculty of Rehabilitation Sciences, Tehran University of Medical Sciences (TUMS), Tehran, Iran*; 2 *Brain & Spinal Cord Injury Research Center, Imam Khomeini Hospital Complex, Tehran University of Medical Sciences (TUMS), Tehran, Iran*; 3 *Digestive Disease Research Institute (DDRI), Shariati Hospital, Tehran University of Medical Sciences (TUMS), Tehran, Iran*; 4 *Department of Physical Medicine and Rehabilitation, Neuromusculoskeletal Research Center, Firoozgar Hospital, Iran University of Medical Sciences (IUMS), Tehran, Iran *; 5 *Department of Physical Medicine and Rehabilitation, Firoozgar Hospital, Iran University of Medical Sciences (IUMS), Tehran, Iran*

**Keywords:** Constipation, Validity, Reliability, Persian version, Questionnaire

## Abstract

**Aim:**

The present study aimed to cross-culturally adapt and assess the validity and reliability of the English version of the Wexner questionnaire translated into the Persian language in Iranian patients.

**Background:**

Constipation is one of the most common gastrointestinal disorders; therefore, it is necessary to utilize an index for both the clinic and research studies.

**Methods:**

In the first phase, the English version of the Wexner questionnaire was translated into the Persian language. In the second phase, the Persian version was assessed to evaluate the psychometric properties in 136 patients with functional constipation who referred to gastrointestinal and physical medicine clinics. Content validity was assessed by face validity. Construct validity was tested based on hypothesis testing and structural validity. The correlation of the total scores of the Wexner questionnaire and the Patient Assessment Constipation Quality Of Life (PAC-QOL) questionnaire was used for concurrent criterion validity. Internal consistency and test-retest reliability were calculated using Cronbach's α and intraclass correlation coefficient (ICC).The floor/ceiling effect of the questionnaire was also evaluated.

**Results:**

The content validity of the Persian version of Wexner’s questionnaire was acceptable. The construct and concurrent criteria validity showed moderate correlation. The internal consistency and intrarater reliability were moderate (0.51) and excellent (r_p_ = 0.97, *p*-value <0.001), respectively. No floor/ceiling effect was seen.

**Conclusion:**

The Persian version of the Wexner questionnaire showed good validity and reliability in Iranian patients and can therefore be applied in clinics as well as in research for Persian-speaking countries.

## Introduction

 Constipation is a symptom-based complaint when defecation is unsatisfactory in a multidimensional concept. It consists of infrequent defecation, the sensation of incomplete evacuation, and anorectal blockage during defecation. Painful evacuation, bloating and abdominal pain, excessive straining, enema, and manual maneuver for extracting stool are also among the complaints of these patients (1-3). Constipation is classified into the two subtypes functional and structural according to its causes. Based on the criteria of the current research, this study focused only on functional constipation which includes slow transit constipation (STC) and outlet dysfunction. STC is deﬁned as delayed transit throughout the colon. Outlet dysfunction is an abnormality in the anorectal mechanism wherein the pelvic floor muscles fail to relax or have a paradoxical contraction during defecation or an inadequate increase in rectal or intraabdominal pressure during attempts at defecation (4-7).

Constipation is a common disorder all over the world. Its prevalence in North America is reported to range from 3.1% to 31%, but in Europe and Australia it is estimated to be 17.1% and 24%, respectively; in Asia, the prevalence is reported to be 2.4% to 28.4% (2, 8, 9). In Iran, a review article published in 2012 reported the prevalence of constipation as ranging from 1.4% to 37% and that of functional constipation as ranging from 2.4% to 11.2% (10). However, in a systematic review and meta-analysis article, the prevalence in the adult population of the world ranged from 2.4% to 39.6 with an average of 14%. This disagreement could be due to a variety of reasons, including various definitions of constipation, different data collection methods, and different criteria in epidemiological studies (8, 11).

To assess and evaluate constipation, some paraclinical tests and questionnaires are available. Among them, Wexner is a self-report questionnaire commonly used to assess the severity and prevalence of constipation. It was collected according to more than 100 symptoms related to constipation and is well correlated with the physiologic findings of constipated patients (anorectal manometry, colonic transit time, defecography, electromyography) (12, 13). As Persian is the most common language spoken in Iran, Afghanistan, Tajikistan, and some parts of Iraq and Pakistan (14), the purpose of this study was to cross-culturally adapt and assess the validity and reliability of the questionnaire in Persian-speaking countries. 

## Methods

**First phase: Translation process**

Beaton's guidelines were used to forward translate the English version of the Wexner questionnaire into the Persian language. The questionnaire was independently translated by two experts; one of them was a gastrointestinal specialist and the other was an expert in the Persian language. Afterward, the translators and the main researcher compared and matched the two translated versions, resolving disagreements and synthesizing the Persian version of the questionnaire. In the next stage, the questionnaire was back translated by two native English speakers and linguistic experts. The English version of the questionnaire was blinded to them, and they were asked not to search the original English version. Then, an expert committee, comprising all of the translators, the main researcher, one gastroenterologist, and a biostatistician, reviewed all the disparities and approved the final version of the questionnaire. Accordingly, a pilot study was performed to assess the feasibility of the questionnaire, and then the psychometric properties of the Persian version of the Wexner questionnaire were evaluated for the patients (15).

**Second phase: Validity and reliability**

**Participants**

This was a cross-sectional test development study in which one hundred and thirty-six patients were recruited by a non-random convenience sampling method to evaluate the validity and reliability of the Persian version of the Wexner questionnaire. Participants were from among patients referring to the Gastrointestinal Clinic of Shariati Hospital (TUMS) and the Physical Medicine Clinic in Firoozgar Hospital (IUMS).

Participants were selected based on the following inclusion and exclusion criteria. Inclusion criteria: Age over 20 years, diagnosis of functional constipation according to Rome III criteria and examination of anorectum by a gastroenterologist, no history of neurodegenerative diseases (i.e. multiple sclerosis, spinal cord injury, or stroke), no history of metabolic disorders (i.e. diabetes, hypothyroidism, or hypercalcemia), no history of opioid-induced constipation, no history of irritable bowel syndrome, no history of congenital anorectal abnormalities, no history of colon cancer, no pregnancy, and the ability of reading and writing in the Persian language. Exclusion criteria: unwillingness to continue or having any complaints like bleeding from the anorectum during the test-retest interval. 

Thirty-two patients were chosen to assess the construct and concurrent criterion validity; they completed the Patient Assessment of Constipation Quality Of Life (PAC-QOL) questionnaire at the same time they completed the Wexner questionnaire.Forty-three patients were retested by the questionnaire after two weeks to assess intrarater reliability; these patients did not receive any medical intervention and maintained their diet and usual regimen (16). This study was approved by the Ethics Committee of TUMS (IR.TUMS.FNM.REC.1396.3299).

**Instruments**

The Wexner questionnaire was developed in 1996 by Dr. Wexner and has been mentioned in articles such as Constipation Scoring System (CSS), 1996 Cleveland Clinic Score, and Agachan Score (12). It includes 8 items that are graded on a five-point Likert scale except for one, which is graded from 0 to 2. The first item is the frequency of bowel movements, for which a score of 0 means "1-2 times per 1-2 days", a score of 1 means "2 times per week", a score of 2 means "once per week", a score of 3 means "less than once per week", and a score of 4 means "less than once per month". Painful evacuation efforts, feeling incomplete evacuation, and abdominal pain are the second, third, and fourth items, respectively, for which scores of 0 to 4 mean "never", "rarely", "sometimes", "usually", and "always", respectively. The fifth item is the time (minutes) spent in the lavatory per attempt for which a score of 0 means "less than 5 min", a score of 1 means "5-10 min", a score of 2 means "10-20 min", a score of 3 means "20-30 min", and a score of 4 means "more than 30 min". The sixth item is the type of assistance for which a score of 0 means "without assistance ", a score of 1 means "stimulative laxatives", and a score of 2 means "digital assistance or enema". The seventh item is unsuccessful attempts for evacuation per 24 hours for which scores of 0 to 4 mean "never ", "1-3", "3-6", "6-9", and "more than 9", respectively. The eighth item is the duration of constipation (yrs.) for which scores of 0 to 4 mean "0 (yrs.)", "1-5 (yrs.)", "5-10 (yrs.)", "10-20 (yrs.)", and "more than 20 (yrs.)", respectively. Based on the grading of the questionnaire, scores range from 0 (normal) to 30 (severe constipation), and a score of 15 is the cut-off point (12, 13).

The PAC-QOL was developed by Marquis et al. to investigate the effects of constipation on patients' quality of life over 2 weeks. It includes 28 items in 4 dimensions consisting of physical discomfort (4 items), psychosocial discomfort (8 items), worries and concerns (11 items), and treatment satisfaction (5 items). Questions are scored based on a Likert scale of 0 (not at all/none of the time) to 4 (extremely/all of the time). The higher the patient's score is, the greater the negative effects of constipation are on the patient's quality of life (17-19). The validity and reliability of the PAC-QOL questionnaire in Iran were examined in 2017 and 2018 in two separate studies. In the study of Mokhtare (2017), content validity ratio (CVR) ranged from 0.5 to 0.8, and the value of the content validity index (CVI) was 0.81 for content validity. Moreover, Cronbach's α (0.975) and intra-class correlation coefficients (0.974) were reported for internal consistency and reliability, respectively (19).

**Validity and reliability**

The psychometric properties of the Wexner questionnaire were determined and are presented below.

**Content validity**

Content validity examines how the items of an instrument demonstrate the concepts that is to be measured (16). In this study, the Wexner questionnaire was translated and adapted, so only the face validity was assessed. Face validity measured the appropriateness of the tool appearance and was evaluated by a quantitative form with an impact score. The impact score was calculated using a 5-point Likert scale. Five gastroenterologists with experience in the diagnosis and treatment of constipation were asked about each item. Then the formula Frequency × Importance was used. Frequency means a percentage of experts who scored the items 4 or 5, and the importance of an item was calculated by the mean value of items. On the Likert scale, a score of 5 means "very important", 4 means "important", 3 means "relatively important", 2 means "slightly important", and 1 means "unimportant". Items with an impact score of greater than 1.5 were retained; all others were excluded (20, 21). 

**Construct validity**

The construct validity was measured by hypothesis testing and structural validity. Two hypotheses were considered, as follows (16):

1) A moderate positive correlation between the total score of the Wexner questionnaire and the scores of "physical discomfort", "psychological discomfort", and "worries and discomfort" as the subscales of the PAC-QOL questionnaire (convergent validity).

2) No correlation between the total score of the Wexner questionnaire and the score of the "treatment satisfaction" subscale.

Structural validity was evaluated through factor analysis which was performed to determine the possible dimensions of the Wexner questionnaire. As there are no assumptions about the dimensions, a principal component analysis (PCA) was performed based on eigenvalues greater than 1. The extracted factors were rotated with the Varimax method. Factor loading was accepted at the ≥0.40 level. To control the missing data, the researchers ensured that participants accurately completed all items in the questionnaire (22). 

**Concurrent criterion validity**

As no gold standard was available for comparison, the total score of the Wexner questionnaire was compared with the total score of the PAC-QOL questionnaire (16). 

**Reliability**

Internal consistency reliability was assessed by Cronbach's alpha, and a value equal to or greater than 0.7 was considered acceptable (16). The reliability of the Wexner questionnaire was evaluated by the test re-test method. An intra-class correlation coefficient (ICC) ( ≥ 0.7) was considered acceptable (16). Paired t-tests were also employed to test the reliability of each item and the total score of the Wexner questionnaire.

**Floor/ceiling effect**

The presence of ﬂoor or ceiling effects indicates limited content validity and reduced reliability. A value of more than 15% of participants who attained a total score higher than 80% or lower than 20% of the Wexner questionnaire was considered as the effects (16). 

**Statistical analysis**

Mean ± SD was used for descriptive data (SPSS software version 20), and the Kolmogorov-Smirnov test was performed to check normal distribution in quantitative variables. The significance level was defined using alpha <0.05. To determine the construct validity and concurrent criterion validity, Pearson’s correlation coefficient was used. The coefficient lower than 0.40 indicated a weak, between 0.40 and 0.70 a moderate, and greater than 0.70 a strong correlation.

## Results

One hundred and thirty-six patients with functional constipation were enrolled in the current study. Participants had a mean (SD) age of 42.47 (14.21) years; 79.4% were female and 20.6% were male, and 77.6% of them were married.

**Content validity**

**Table 1:** Pearson's correlation coefficients between the total score of the Wexner questionnaire and each of the items and the total score of the PAC-QOL questionnaire

	Total score of the Wexner questionnaire	
	Correlation coefficient	P-value
Physical discomfort	0.478	0.006**
Psychological discomfort	0.406	0.021*
Worries and discomfort	0.440	0.012*
Treatment satisfaction	0.238	0.189
The total score of PAC-QOL	0.529	0.002**

*Correlation is significant at the 0.05 level; **Correlation is significant at the 0.01 level

**Table 2:** Values of total initial eigenvalues and percentages of variance

	Total initial eigenvalues	% of the variance (extraction sums)	% of the variance (rotation sums)
Component 1	1.963	24.542	19.994
Component 2	1.440	17.994	18.861
Component 3	1.052	13.156	16.839

The mean impact score for all items was 4.64 (ranges from 3.36 to 5). Thus, all of them had an acceptable score in face validity.

**Construct validity**

The total score of the Wexner questionnaire showed a moderate correlation with "physical discomfort", "psychological discomfort", and "worries and discomfort" as subscales of the PAC-QOL questionnaire. Furthermore, there was no significant correlation between the total score of Wexner questionnaire and the "treatment satisfaction" subscale (Table 1). 

Factor analysis by varimax rotation recognized three components with eigenvalues greater than 1 and factor loading ranged from 0.6 to 0.7, and detected 55.69% of the total variance. The factor loadings were classified as the first component including 3 items (items 1, 7, and 8), the second component including 3 items (items 2, 3, and 4), and the third component including 2 items (items 5 and 6) (Table 2).

**Concurrent criterion validity**

The results showed the Wexner questionnaire score was significantly correlated with the PAC-QOL questionnaire's total score (*p*-value = 0.002), and Pearson's correlation coefficient was 0.52, indicating a moderate relationship between them (Table 1).

**Reliability**

Cronbach's alpha coefficient was 0.51 for all items, indicating a moderate internal consistency**.** The test-retest of the total score with ICC (two times and one examiner) was excellent (r_p_ = 0.97, *p*-value<0.001). The paired t-test showed no significant difference between any of the items and the total score of the Wexner questionnaire (*p*-value = 0.232, 95% CI: − 0.12 to 0.49) on tests.

**Floor/ceiling effect**

There was no floor/ceiling effect for the questionnaire. Only 3.67% of participants attained a score lower than 20% and 1.47% attained a score higher than 80% of the total score of the Wexner questionnaire.

## Discussion

The Wexner Questionnaire is a common tool for examining the severity of constipation in research as well as in the clinic. It is an easy, clear, well-communicative, and feasible tool for patients, although it has not been formally validated (23). In the current study, the translation and validation procedures showed content retention, moderate construct and concurrent criterion validity correlation, moderate internal consistency, and excellent intrarater reliability. Thus, the translated version can be used as a standard tool in clinics and research in Persian-speaking countries. No particular problems were encountered in this process, as the gastroenterologists collaborated and interacted excellently in clarifying the questionnaire items. The items were comprehensible to the participants, and there was no missing data. Participants answered all items in less than 5 minutes, which may demonstrate that items were clear and acceptable.

To the best of our knowledge, no study is available in the literature about the Wexner questionnaire for constipation. However, two studies in Brazilian and Turkish societies have validated and adapted the Wexner questionnaire for fecal incontinency; therefore, those results could not be compared with the current ones (24, 25).

**Content validity**

The content validity of the questionnaire demonstrated a high score using the impact score. During the preparation of the questionnaire, items were precisely checked by gastroenterologists and researchers to get clear and transparent concepts. 

**Construct validity**

As mentioned earlier in the results section, a significant correlation was seen between the total score of the Wexner questionnaire and the score of all subscales of the PAC-QOL except for the "treatment satisfaction" subscale. Forasmuch as their correlation coefficient was moderate, we attribute this value to the fact that the Wexner questionnaire items were somewhat different from the subscales of the quality of life questionnaire. The participants in this study had received a treatment prior to entering the current study, so this variable showed no significant correlation in the "treatment satisfaction" subscale. Tsunoda et al. compared subscales of PAC-QOL with the Wexner questionnaire and found correlations of 0.05 (treatment satisfaction subscale) to 0.27 (overall score) (26), which differ somewhat from the current results. This discrepancy could be due to the differing study design or the different inclusion and exclusion criteria of these two studies (i.e. the current study included functional constipation, while theirs included chronic constipation).

PCA was used to evaluate factor analysis, and the results showed three components with a total variance of 55.69%. Consequently, we suggest considering terms of "assessment of defecation frequency" for the first component including 3 items (items 1, 7, and 8), "abdominal and evacuation discomfort" for the second component including 3 items (items 2, 3, and 4) and "difficulty when visiting the lavatory" for the third component including 2 items (items 5 and 6). However, as the total variance of the exploratory factor analysis results did not show a large value, it is worth considering the use of the questionnaire consisting of eight distinct dimensions, with each dimension representing one of the symptoms of constipation disorder, in a future study.

**Concurrent criterion validity**

A significant and moderate correlation was seen between the total score of the Wexner questionnaire and the total score of the PAC-QOL questionnaire. Due to the lack of a gold standard for comparing the Wexner questionnaire, the total score of the questionnaire was compared with the total score of the PAC-QOL questionnaire. The Wexner questionnaire evaluates the severity of symptoms in constipated patients, whereas, the PAC-QOL questionnaire examines the effects of constipation on various aspects of life. The current results were in line with the results of the Bengi et al. study in which the concurrent validity of the PAC-QOL questionnaire was compared with the Wexner questionnaire (correlation coefficient = 0.457 and *p*<0.001) (27).

**Reliability**

The internal consistency of the instrument was moderate, so it may be concluded that each of the items in this questionnaire examines a different aspect of constipation symptoms (16). The Persian version of the Wexner questionnaire indicated excellent interarater reliability, which could represent that the results of the questionnaire were consistent over time, and therefore, any change in symptoms can be interpreted as a reliable outcome measure.

**Floor/ceiling effect**

The results indicated that items on the Wexner questionnaire could represent the content that was reliable for assessing the severity of constipation symptoms. 

The first limitation was the lack of assessment of diagnostic validity and non-determination of the cut-off point for patients with constipation in Persian-speaking countries. Therefore, these items should be considered in future studies. Second, only functional constipated patients participated in this study; therefore, a study to apply the questionnaire in other subtypes of constipation is required. Third, women experience more pelvic floor dysfunctions (e.g., through pregnancy and delivery) than men; therefore, the percentage of women was higher than men in the present study. Accordingly, an equal composition of men and women is suggested for a future study. 

The Persian version of the Wexner questionnaire can be applied in both clinical practice and research in Persian-speaking countries. This questionnaire has excellent repeatability, and each item of it can assess a separate issue of constipation symptoms (See appendix).
